# Proof-of-concept for a randomized factorial design strategy to build a complex online support program for cancer survivors with psychiatric symptoms: the IN-FACT-0 feasibility trial

**DOI:** 10.1007/s11764-025-01905-2

**Published:** 2025-10-25

**Authors:** Julia Winter, Nils Gasslander, Gustaf Rane, Felicia Sundström, Linda Björkhem-Bergman, Christel Hedman, Erland Axelsson

**Affiliations:** 1 Department of Molecular Medicine and Surgery, Karolinska Institutet, 171 76 Stockholm, Sweden; 2 R & D Department, Stockholms Sjukhem Foundation, Stockholm, Sweden; 3 Division of Clinical Psychology, Department of Psychology, Uppsala University, Uppsala, Sweden; 4 Division of Psychology, Department of Clinical Neuroscience, Karolinska Institutet, Stockholm, Sweden; 5 Division of Clinical Geriatrics, Department of Neurobiology, Care Sciences and Society, Karolinska Institutet, Stockholm, Sweden; 6 Department of Clinical Sciences Lund, Lund University, Lund, Sweden; 7 Division of Family Medicine and Primary Care, Department of Neurobiology, Care Sciences and Society, Karolinska Institutet, Stockholm, Sweden; 8 Liljeholmen University Primary Health Care Centre, Academic Primary Health Care Centre, Region Stockholm, Stockholm, Sweden

**Keywords:** Cancer survivors, Fear of recurrence, Feasibility studies, Proof of concept study, Telemedicine

## Abstract

**Purpose:**

Internet-delivered cognitive behavioral therapies and lifestyle interventions are promising for reducing psychiatric symptoms in cancer survivors, but little is known about the contribution of specific treatment components to observed effects. A randomized factorial trial could allow for evaluation of the unique contribution of treatment components to overall treatment effects. The IN-FACT-0 trial assessed the feasibility of such an approach for building an online support program for reducing long-term psychiatric symptoms in cancer survivors.

**Methods:**

In a full factorial experiment, 40 survivors of breast, testicular, or thyroid cancer with clinically significant depressive symptoms, anxiety, or fear of recurrence were randomized to combinations of three treatment components: (i) behavioral activation, (ii) systematic exposure, and (iii) healthy lifestyle promotion. All groups, including a control group, received rudimentary support and kept a symptom diary.

**Results:**

On average, therapists rated the feasibility of the study design 9 on a scale from 0 (“not feasible at all”) to 10 (“fully feasible”). The Credibility/Expectancy Scale mean was 36.6 (SD = 8.2). Participants initiated a mean of 6.1 (SD = 2.2, median = 7) out of 8 modules, and 90% (36/40) completed the post-treatment assessment. The mean Client Satisfaction Questionnaire score was 24.3 (SD = 5.7). One adverse event each was reported by 2/36 (6%) participants. No event was serious. The mean reduction was large in depressive symptoms (*d* = 0.83) and moderate in general anxiety (*d* = 0.55) and fear of recurrence (*d* = 0.54).

**Conclusions:**

This randomized factorial trial design was feasible.

**Implications for Cancer Survivors:**

This trial design may be used to develop effective, evidence-based interventions for cancer survivors with psychiatric symptoms.

**Trial registration:**

ClinicalTrials.gov NCT06046573 (registered 2023–09-21).

**Supplementary Information:**

The online version contains supplementary material available at https://doi.org/10.1007/s11764-025-01905-2.

## Background

 The experience of cancer is associated with a range of challenges that may lead to psychiatric symptoms and reduced health-related quality of life [1]. Examples of challenges include a heightened awareness of death [2], unwanted changes in physical appearance or bodily functions such as capacity for intimacy [3], fatigue [4], traumatic experiences during the healthcare process [5], and adverse effects of cancer treatment [6, 7]. Most studies indicate that clinically relevant depression and anxiety are common in cancer survivors, with some cancer subtypes reporting prevalences over 40% [8]. More than half of cancer survivors experience at least a moderate fear of cancer recurrence over a decade after diagnosis [9] and managing fear of recurrence is frequently reported as an unmet need by cancer survivors [10]. Fear of cancer recurrence persists over time; in the year following cancer diagnosis, around one in five cancer survivors experience fear of recurrence at levels indicating a need for specialized intervention, and this proportion persists over 10 years after diagnosis [9]. Long-term psychiatric symptoms are also common in patients with cancers typically considered favorable, such as breast, thyroid, and testicular cancer [11]. Paradoxically, despite being likely to live with their psychiatric symptoms for a substantial period of time, these patients may be overlooked in the clinical setting due to clinicians perceiving them as suffering from a “good” cancer [12]. Heightened psychiatric symptoms predict both mortality [13] and excess disease burden [14] and are identified as a top priority for intervention by cancer survivors [15].

Cognitive behavioral therapy (CBT) is the most researched psychological intervention framework for psychiatric symptoms in cancer survivors [16, 17]. CBT is effective in improving psychological symptoms such as depression and anxiety in cancer patients [18, 19], as well as reducing fear of cancer recurrence [20, 21]. CBT in combination with healthy lifestyle interventions has also been found to improve quality of life and psychological symptoms in cancer survivors [22]. Administering interventions online increases access to treatment, and the effects of cognitive behavioral therapies are typically similar to face-to-face therapy [23]. Online psychological interventions can improve long-term psychiatric symptoms after cancer, and multicomponent protocols have shown particular promise [24, 25]. Such multicomponent protocols have commonly been based on components such as behavioral activation, cognitive restructuring techniques, exposure- or behavioral experiment-based strategies, mindfulness-based strategies, the promotion of coping strategies, problem-solving, psychoeducation, relaxation, and self-management strategies [24, 26–28]. However, it is not known to what degree these treatment components contribute to overall effects, or how components are best combined.

By evaluating the effect of individual treatment components and their combinations, it is possible to optimize psychological interventions to be more effective and efficient. For this purpose, a factorial experiment may be employed [29]. In a randomized factorial trial, each study participant may be assigned more than one property by means of randomization [30]. That is, instead of assigning each participant to *one* condition (a or b), each participant is assigned to a combination of conditions (e.g., a, b, c, a + b, a + c, b + c, or a + b + c). An important advantage of factorial experiments is that the contribution of individual treatment components and their combinations to the effect of an overarching, multicomponent treatment can be efficiently evaluated in one clinical trial. In this proof-of-concept study, we aimed to assess the feasibility of such a randomized factorial study design to evaluate the unique contribution of specific treatment components to the overall efficacy of an internet-delivered treatment for long-term psychiatric symptoms in cancer survivors.

## Methods

### Design

The IN-FACT-0 trial was a pilot study that evaluated the feasibility of a randomized factorial study design for the experimental evaluation of specific treatment components and their contribution to the overall effect of an online psychological treatment for psychiatric symptoms in cancer survivors. The trial was based at Karolinska Institutet, Stockholm, Sweden. The study was performed in line with the principles of the Declaration of Helsinki, and the protocol was approved by the Swedish Ethical Review Authority (2023–04056−01). The trial was preregistered at ClinicalTrials.gov on September 21, 2023 (NCT06046573). The original target sample size was 48, which was revised to 40 (see Online Resource 1 for a description of the reason for this change). Outcomes are reported according to the 2010 CONSORT extension to randomized pilot and feasibility trials [31].

### Recruitment and participants

The target population was adult cancer survivors treated for breast, testicular, or thyroid cancer, with main treatment (surgery, radiotherapy, chemotherapy, immunotherapy, radioiodine) ending at least 6 months ago, without a medical status that would make the support program inappropriate, who report clinically relevant mild to moderate psychological distress, and who are interested in working with a structured online support program. There was no upper time limit to the duration from the end of cancer treatment; something that can be summarized as a “primary care approach to cancer survivorship,” where a broad and heterogeneous group of survivors report problems that they attribute to survivorship long after receiving their diagnosis. Participants with breast, testicular, and thyroid cancer were selected due to studies showing that psychological symptoms are common among patients with these cancers, despite a favorable prognosis [11, 32, 33]. Furthermore, psychological needs are the most frequently reported unmet supportive care need in these patients [34–36]. The main rationale for including a heterogeneous study population with regard to cancer type, time since cancer treatment, and symptom profile was that mental healthcare resources are scarce in both primary care and oncology, and therefore, under realistic routine care scenarios (regardless of the precise format of administration, for example, book form, webpage, app, or a few face-to-face meetings), any form of psychiatric assessment and therapist support would probably have to be limited, and providing a large number of support programs for different types of cancer-related psychological distress is typically not realistic. This speaks for broad interventions built on widely tested strategies that can be easily disseminated in many formats and settings with minimal resources, and then easily tailored by the patients themselves. Heterogeneity will also enable analyses of moderators and mediators of treatment effects in a future full-scale trial [37, 38], including the possibility of testing for conditional indirect effects, i.e., if the relevance of mediators (such as the importance of increasing physical activity, or reducing symptom preoccupation) differs as a function of patient baseline characteristics [39, 40].

Information about the study was posted on social media, the Karolinska Institutet website, and through patient organizations for breast cancer (Bröstcancerföreningen Amazona), thyroid diseases (Sköldkörtelförbundet), and for young patients with cancer (Ung Cancer). The heading of the posting was “Is your wellbeing affected after cancer?” and the posting specified that the study was aimed at individuals treated for breast, testicular, and thyroid cancer who would like more support. Participants of an ongoing cohort study of thyroid cancer who had disclosed that they were interested in psychological treatment were also informed about the trial by letter. All study information directed interested individuals to apply via the study website.

Applicants provided informed consent through a secure web form and then completed a screening that included validated questionnaires and collected sociodemographic and clinical characteristics. Applicants who did not fulfill the eligibility criteria at screening (see below) were informed and referred to routine healthcare services through a standardized email. Brief structured telephone interviews were conducted with the remaining applicants to establish eligibility. These were held by a licensed physician or clinical psychologist and were primarily based on the Mini-International Psychiatric Interview version 7 [41], with a separate module for clinically significant health﻿ anxiety (available at OSF.io, identifier: t3yvz) [42].

Inclusion criteria were as follows: (i) survivor of breast, testicular, or thyroid cancer with no evidence of remaining disease or recurrence; (ii) at least 6 months after main cancer therapy (surgery, radiotherapy, chemotherapy, immunotherapy, radioiodine); (iii) clinically significant anxiety (Generalized Anxiety Disorder 7 [GAD-7] ≥ 8), depressive symptoms (Patient Health Questionnaire 9 [PHQ-9] ≥ 10), or fear of cancer recurrence (9-item Fear of Cancer Recurrence Inventory [FCRI-9] ≥ 16); (iv) age ≥ 18 years; (v) resident of Sweden (listed and de facto); (vi) sufficient technical and Swedish language knowledge to take part in a text-based treatment online; (vii) continuous access to an electronic device to access the study web platform; and (viii) complete pre-treatment assessment. We aimed to recruit 24 survivors of breast cancer, 12 survivors of testicular cancer, and 12 survivors of thyroid cancer; see Online Resource 1 for details. We selected a 6-month post-treatment eligibility to avoid the turbulent time around diagnosis and treatment when patients may be experiencing acute psychological distress and side effects of treatment that would make a psychological intervention of this format inappropriate. Exclusion criteria were as follows: (i) recurrent thoughts of suicide based on clinical assessment aided by the structured interview and the self-report version of the Montgomery-Åsberg Depression Rating Scale (MADRS-S) item 9; (ii) severe psychiatric or medical condition, medical treatment, or ongoing diagnostic workup that would make the study treatment unfeasible; (iii) other ongoing psychological treatment; (iv) continuous psychotropic medication not stable for the past 4 weeks or not expected to remain stable during the treatment period; and (v) planned absence longer than 1 week of the treatment period. The exclusion criteria for severe psychiatric condition was not based on any particular diagnosis but rather an overall assessment of symptom severity, need for continuous medical attention, frequency and intensity of suicidal ideation, and functional impairment in accordance with standard practice of the Swedish healthcare system; typical examples warranting referral to specialist psychiatry include a psychotic disorder or severe depression. A severe medical condition was considered to be any medical condition with very poor prognosis, such as metastatic cancer.

### Internet-delivered factorial cancer support program

A therapist-guided internet-delivered treatment was administered over 10 weeks. Content was conveyed through illustrated texts, presented as eight consecutive modules. Each module contained assignments and questions for reflection. Access to new modules was given contingent on progress. Participants communicated with a therapist via an email-like system and could expect a reply within around 2 working days. To encourage adherence, therapists attempted to contact participants who had been inactive on the platform for more than a few days. Therapists received weekly and on-demand supervision by a licensed psychologist to promote competence and adherence. See Online Resource 2 for more information about the study therapists.

Variations of the internet-delivered treatment were assigned by the combination of specific treatment components in a full factorial experiment. Randomization relied on a true random number service (www.random.org) and was conducted by an individual otherwise not involved in the project, blind to participant characteristics, and after the decision to include the participant in the study. Each randomized cohort had a size that was a multiplier of eight—the number of unique treatment component combinations, to which participants were equally allocated. The study design was partially blinded. Participants were informed that they would participate in an online support program for improved wellbeing after cancer but were not aware of the factorial nature of the study.

The following three treatment components (factors) were used: (a) behavioral activation with existential themes and an emphasis on values and relationships; (b) systematic exposure with response prevention with a focus on anxiety about health, death, body image, and cancer-related trauma; and (c) healthy lifestyle promotion focusing on physical exercise, dietary advice, and strategies for improved sleep. The treatment components of behavioral activation and systematic exposure were chosen due to being among the most commonly used components of CBT in behavioral medicine and in the psychological treatment literature as a whole. Behavioral activation is the most widely evaluated cognitive-behavioral strategy for reducing depressive symptoms [43]. Exposure-based strategies with mindfulness training can be effective in reducing symptom preoccupation and various forms of health anxiety, which, to a certain extent, can be considered to overlap with fear of cancer recurrence [44–47]. The systematic exposure component in this study therefore also contained elements of mindfulness training with an exercise to unconditionally observe one’s body and advice for simply observing and not acting on catastrophic thoughts. Healthy lifestyle interventions that address health behaviors such as physical activity and healthy eating are also broadly used to improve health and well-being, including improving quality of life in cancer survivors [48, 49].

**Fig. 1:**
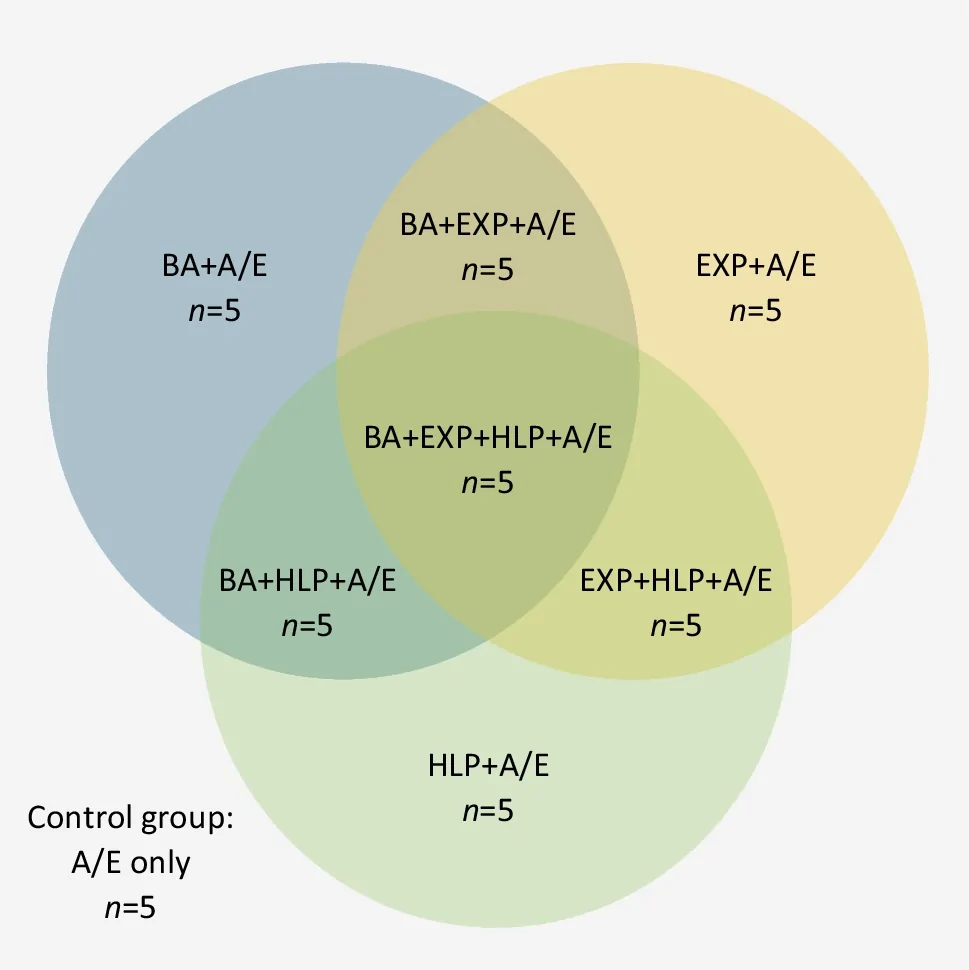
Randomized factorial study design based on the experimental manipulation of treatment components of an online support program for negative long-term psychiatric effects of cancer. Five participants were randomized to each unique combination of the following three treatment components: behavioral activation (BA), systematic exposure (EXP), and healthy lifestyle promotion (HLP), including a control group that did not receive any of these three treatment components. All participants, including the control group, logged in regularly to the online support platform and received rudimentary support from a therapist (attention/engagement, A/E)

The treatment content was developed with input from a patient representative. See Online Resource 3 for descriptions of the treatment content. Five participants were randomized to each unique combination of treatment components, including a control group randomized to receive none of the three aforementioned components (in total, *n* = 40) (Fig. 1). All groups, including the control group, were given rudimentary information about the long-term effects of cancer, received support from a therapist, had the opportunity to set personal goals, and were encouraged to track and report their wellbeing. Such elements of attention/engagement often explain a substantial proportion of the effects of psychological interventions [50]. For ethical reasons, after the 10-week treatment, participants in the control group were offered to be crossed over to the treatment variant that had all components.

### Measures

Key feasibility outcomes were (1) overall feasibility as rated by therapists, (2) patient-reported credibility, (3) adherence to the protocol, (4) acceptability of the measurement strategy, (5) patient-reported satisfaction, (6) rates of adverse events and negative experiences, and (7) within-group improvement of general anxiety, depressive symptoms, fear of recurrence, and health-related quality of life.

Online self-report questionnaires were administered through the study web platform at screening, pre-treatment, every week during treatment, post-treatment, and 3 months after treatment. Participants in the control group, having been crossed over, did not complete the three-month follow-up.

Depressive symptoms were assessed using the PHQ-9 [51], general anxiety using GAD-7 [52], and fear of cancer recurrence using FCRI-9 [53] at all assessment points. Health-related quality of life was measured before and after treatment using the 36-item Short Form Health Survey v2.0 [54]. 

Treatment credibility and relationship with the therapist were assessed at week two of the treatment using the Credibility/Expectancy (C/E) Scale [55] and the Working Alliance Inventory (WAI) [56], respectively. Adherence was measured in terms of the number of modules initiated, as well as by questions tailored to each treatment arm at each weekly assessment. These questions asked about the number of (a) days devoted to activity planning, (b) completed mindfulness and exposure exercises, and (c) days devoted to the promotion of health behaviors in the past 7 days. At the post-treatment assessment, participants rated their adherence during the first and second halves of the treatment period using 5-item Likert scales with items ranging from “have not followed the treatment at all” to “have followed the treatment completely”.

Satisfaction with the treatment was evaluated at the post-treatment assessment using the 8-item Client Satisfaction Questionnaire (CSQ-8) [57]. Additionally, participants rated their satisfaction with each treatment component, except for attention/engagement, using a rating scale from 0 to 10, where 0 was “not at all beneficial and useful” and 10 was “extremely beneficial and useful.” Negative effects and adverse events were assessed post-treatment using the 20-Item Negative Effects Questionnaire (NEQ-20) [58], as well as a questionnaire regarding adverse events, previously used in other clinical trials [45, 59, 60]. In this questionnaire, participants are asked, using free text items, whether they experienced any adverse events that they are then asked to describe and rate the effect of. Participants were also asked to rate the strain associated with the measurement strategy from 0 (“not at all stressful or burdensome”) to 10 (“extremely stressful or burdensome”), and to describe their experience of the treatment and give feedback in a free-text item. Another 12 questionnaires were administered solely for the purpose of evaluating feasibility in terms of the strain of the measurement strategy and are therefore not reported in this article (see Online Resource 4 for a summary of all questionnaires). Therapists were asked to rate the feasibility of the study design and each treatment component from 0 (“not feasible at all”) to 10 (“fully feasible”) within 45 days after the end of treatment.

At the 3-month follow-up assessment, a blinding check was conducted where participants were informed that they had been randomized to different types of support programs. Participants were asked if they knew which these were and whether they knew which types of support programs the researchers thought would be most effective.

### Statistical analysis

Analysis was conducted using SPSS Statistics 28.0.0.0. Feasibility outcomes were reported descriptively, and no inferential statistics were employed. Although not the primary focus of this feasibility study, preliminary efficacy in terms of within-group average symptom change was also analyzed using linear mixed effects regression models under maximum likelihood estimation (up to 100 iterations). These models included a random intercept but not a random slope and were fitted on data from all participants. A piecewise regression approach was used to model distinct rates of change during the treatment and follow-up periods, with a spline at the post-treatment assessment. Within-group standardized effect sizes were calculated as Cohen’s *d*, with the model-implied mean change from the mixed effects models as the numerator and pre-treatment standard deviation as the denominator. A value of *d* = 0.2 was regarded as a small effect, *d* = 0.5 as a moderate effect, and *d* = 0.8 as a large effect [61].

## Results

**Fig. 2:**
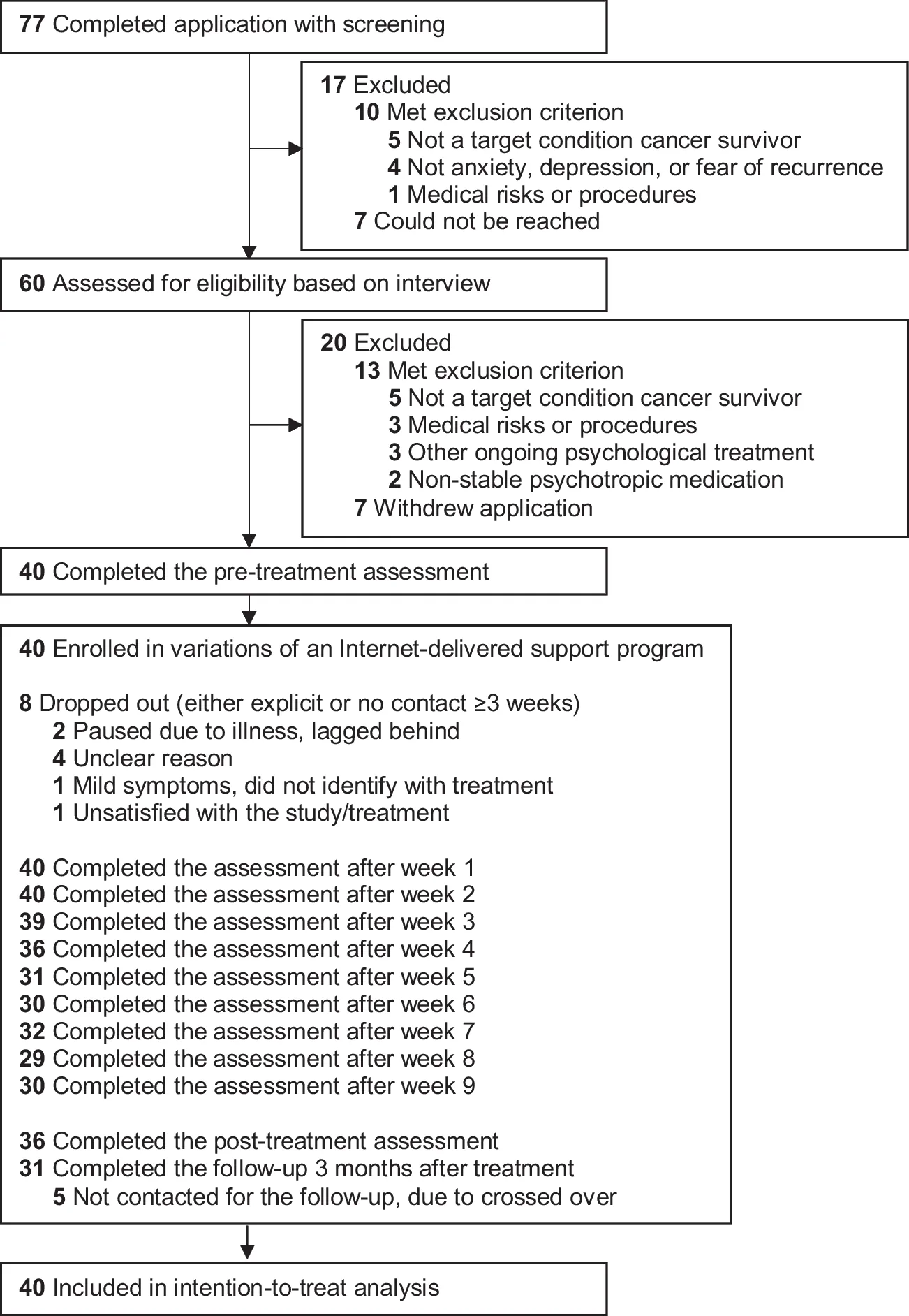
Flowchart showing recruitment and participation

The first participants were enrolled on March 7, 2024, and the last 3-month follow-up was returned on October 22, 2024. A study flowchart is presented in Fig. 2, and sample characteristics are presented in Table 1. A typical participant was around 53 years old (SD = 11), female (37/40, 93%), highly educated, and was treated for breast cancer 40 months previously (SD = 45). Almost all participants (39/40, 98%) experienced clinically significant fear of cancer recurrence, and 58% (23/40) and 45% (18/40) had clinically significant depressive symptoms or anxiety, respectively. The most common psychiatric disorders based on the structured telephone interview were clinically significant health anxiety (20/40, 50%), major depressive disorder (16/40, 40%), and generalized anxiety disorder (7/40, 18%). A quarter of participants (10/40) reported psychotropic medication, and a majority (27/40, 68%) had received previous psychological treatment or other support, such as from a counsellor.

**Table 1:** Characteristics at screening of 40 participants previously treated for breast, thyroid, or testicular cancer

**Sociodemographic characteristics**	
Age	53 (11), 36–76
Female gender	37 (93%)
University education^a^	30 (75%)
Married or de facto	33 (83%)
Has children	36 (90%)
*Employment*	
Working full-time	27 (68%)
Working part-time (< 90%)	2 (5%)
Unpaid work or parental leave	1 (3%)
Unemployed	1 (3%)
Retired	4 (10%)
Student	2 (5%)
Other	3 (8%)
**Clinical characteristics**	
*Type of cancer*^*b*^	
Breast	30 (75%)
Thyroid	10 (25%)
Testicular	2 (5%)
Other	5 (13%)
Months since cancer treatment	40 (45), 5–252 (median 24, IQR=52)
*Psychiatric symptoms*	
Depressive symptoms (PHQ-9)	10 (3), 3–16
Clinically significant depression, PHQ-9 ≥ 10	23 (58%)
Anxiety (GAD-7)	7 (4), 0–18
Clinically significant anxiety, GAD-7 ≥ 8	18 (45%)
Fear of cancer recurrence (FCRI-9)	23 (5), 12–33
Clinically significant fear of recurrence, FCRI-9 ≥ 16	39 (98%)
*Psychiatric diagnoses*^*c*^	
Major depressive disorder	16 (40%)
Clinically significant health anxiety	20 (50%)
Generalized anxiety disorder	7 (18%)
Other anxiety disorder, OCD, or PTSD	4 (10%)
Alcohol or substance use disorder	0 (0%)
*Medication*	
Psychotropic	10 (25%)
Pain (prescribed)	5 (13%)
Previous psychological treatment	15 (38%)
* Previous psychological treatment *via internet	3 (8%)
Previous supportive care, not psychological treatment	12 (30%)
**Recruitment path**	
Through another study	4 (10%)
Through social media	32 (80%)
Friend, acquaintance or family member	2 (5%)
Other	2 (5%)

Estimates are presented as M (SD), range or *n* (%)

^a^International Standard Classification of Education 1997 (ISCED-97) level 4 or higher

^b^Two participants had previously received diagnoses of both breast and thyroid cancer

^c^Based on a structured telephone interview. See Online Resource 5 for more detailed results

*PHQ-9* Patient Health Questionnaire 9, *GAD-7* Generalized Anxiety Disorder 7, *FCRI-9* 9-item Fear of Cancer Recurrence Inventory, *IQR* interquartile range

### Participant-therapist interactions

The mean number of messages sent by participants to their therapist through the email-like system of the study web platform was 6 (SD = 4), and participants received an average of 13 (SD = 4) messages from their therapist. Therapist minutes per participant and week were, on average, 6 min (SD = 5). At least one phone call was made to half of the participants (20/40) from their therapist, and three participants (8%) received more than one phone call (2, 3, and 6 phone calls). The mean WAI score, a measure of therapeutic alliance, at 2 weeks was 28 (SD = 10) out of 42 (Table 2).

**Table 2:** Key feasibility outcomes according to treatment component and number of treatment components in variations of a text-based online support program for reducing long-term psychiatric symptoms in 40 survivors of breast, thyroid, or testicular cancer

Sample	*n*^b^	Therapist feasibility^c^		WAI		C/E scale		Number of modules initiated				Dropped out^e^		CSQ-8		Likert satisfaction^f^	
		M	SD	M	SD	M	SD	M	SD	Sum	%^d^	*n*	%	M	SD	M	SD
**Total**	40	9	1	28.0	9.8	36.6	8.2	6.1	2.2	245	77	8	20	24.3	5.7		
**Total–A/E only**^**a**^	35			28.3	10.0	37.8	7.2	6.2	2.2	217	78	6	17	24.3	5.7		
**BA**	20	9	1	26.9	10.5	39.3	7.0	6.4	2.4	128	80	3	15	24.6	5.7	6.9	2.5
**EXP**	20	7	3	30.7	9.6	38.2	6.4	5.5	2.4	110	69	5	25	25.8	5.3	6.3	2.9
**HLP**	20	9	1	30.2	10.2	37.9	7.6	6.9	1.9	138	86	2	10	24.7	6.3	7.3	2.5
**A/E only**	5	6	1	26.2	8.8	28.4	11.1	5.6	2.3	28	70	2	40	24.5	7.1		
**1 component**	15			24.7	9.8	37.0	8.1	6.1	1.9	91	76	3	20	22.9	5.5		
**2 components**	15			31.4	8.4	36.5	6.4	6.2	2.2	93	78	2	13	24.1	5.5		
**3 components**	5			29.4	13.6	43.8	4.0	6.6	3.1	33	83	1	20	28.4	5.9		

^a^Total sample without attention/engagement participants (control group)

^b^Number of participants at baseline

^c^Feasibility of the trial design in general and each treatment component rated by therapists on a scale from 0 (“not feasible at all”) to 10 (“fully feasible”). All five therapists rated the feasibility of the overall trial design and the behavioral activation component. The feasibility of the remaining components was rated by four therapists, as not all therapists led participants randomized to all components

^d^Percentage of total number of modules initiated. For example, 20 participants receiving the behavioral activation treatment component initiated 80% (128/160) of the total possible modules during the 10 week treatment (eight modules per participant)

^e^Either explicit drop out or no contact ≥ 3 weeks

^f^Satisfaction with individual treatment components rated by participants on a Likert scale from 0 (“not at all beneficial and useful”) to 10 (“extremely beneficial and useful”)

A/E, attention/engagement; BA, behavioral activation; EXP, systematic exposure; HLP, healthy lifestyle promotion; WAI, Working Alliance Inventory; C/E, Credibility/Expectancy Scale; CSQ-8, 8-Item Client Satisfaction Questionnaire; M, mean; SD, standard deviation

### Feasibility outcome 1: therapists’ experience of the randomized factorial design

The five study therapists rated the feasibility of the planned trial design as 9 (SD = 1, median = 9.0, IQR = 2.5) on a scale from 0 (“not feasible at all”) to 10 (“fully feasible”). Using the same rating scale, the mean feasibility rating of the behavioral activation component was 9 (SD = 1, median = 9.0, IQR = 2.0), the systematic exposure component was 7 (SD = 3, median = 8.5, IQR = 4.8), and the healthy lifestyle promotion component was 9 (SD = 1, median = 9.0, IQR = 0.8). The mean feasibility rating of the attention/engagement arm was 6 (SD = 1, median = 6.0, IQR = 1.5). The most frequent comments concerned the lack of clear parameters of what were acceptable therapist actions within the attention/engagement arm, as well as an ethical stress associated with delivering a perceived less active or effective treatment. Comments also raised that the systematic exposure component seemed to be more conceptually difficult for participants to understand and harder to motivate participants to engage, with a need for improved pedagogical and motivating texts and clearer assignments.

### Feasibility outcome 2: participants’ ratings of credibility

At week two, the mean score on the C/E scale, where a higher score is indicative of higher treatment credibility and expectancy, was 36.6 out of 50 (SD = 8.2, median = 38.5, IQR = 11.0; n = 40) (Table 2). Participants not in the attention/engagement arm reported a mean of 37.8 (SD = 7.2), and participants in the attention/engagement arm reported a mean of 28.4 (SD = 11.1).

### Feasibility outcome 3: participants’ adherence to the treatment protocol and blinding

On average, participants initiated 6.1 (SD = 2.2, median = 7, IQR = 4) out of 8 modules (Table 2). In protocols including behavioral activation, participants spent on average 3.6 (SD = 2.1) days per week devoted to activity planning. Participants in treatment variants with systematic exposure conducted a mean of 2.9 (SD = 2.0) mindfulness exercises per week and a mean of 2.4 (SD = 2.7) exposure exercises per week. In treatment arms including promotion of health behaviors, participants devoted an average of 4.1 (SD = 1.9) days per week to living with healthy habits in terms of physical activity, 4.3 (SD = 2.0) days per week in terms of diet and healthy eating habits, and 4.7 (SD = 1.8) days to sleep habits. In a 5-item Likert scale from 0 (“have not follow the treatment at all”) to 4 (“have followed the treatment completely”), participants rated their adherence on average as 2.9 (SD = 1.0) in the first half of the treatment and 2.3 (SD = 1.3) in the second half.

Based on free communication during the treatment, at least two participants (5%) were unblinded to the study design, one of whom was in the control group and therefore did not answer the blinding check at three months. The other unblinded participant remained unidentified. Based on the questionnaire administered after 3 months, 4/31 (13%) were possibly unblinded to the study design (see Online Resource 6 for more detailed results of the post-treatment blinding assessment).

### Feasibility outcome 4: acceptability of the measurement regimen

The proportion of completed assessments (non-missing data) over the pre- to post-treatment main phase was 87% (383/440), the post-treatment assessment completion rate was 90% (36/40), and the follow-up completion rate was 89% (31/35). This was in line with the preregistered target of at least 70% at the post-treatment assessment and follow-up. Average perceived strain caused by the measurement strategy was rated as 3.5 (SD = 3.4, median = 2.5, IQR = 7.0) on a scale from 0 (“not at all stressful/distressing”) to 10 (“extremely stressful/distressing”). Twenty-eight percent (10/36) of participants found the measurement strategy to be very burdensome (rating ≥ 7), which was slightly more than the preregistered target of a maximum of 25%.

### Feasibility outcome 5: participants’ satisfaction with the treatment

At the post-treatment assessment, participant mean score on the CSQ-8, indicative of satisfaction with the treatment, was 24.3 out of 32 (SD = 5.7, median = 24.5, IQR = 11.8; *n* = 36) (Table 2). Without the inclusion of the attention/engagement participants, the mean was almost identical (M = 24.3, SD = 5.7, median = 24.5, IQR = 11.8; *n* = 32). Three out of four participants (27/36; 75%) stated that they would be likely to recommend the treatment to a friend with similar problems. Without the attention/engagement participants, this proportion did not change (24/32; 75%). On the rating scale measuring satisfaction from 0 to 10, participants rated satisfaction with the behavioral activation component as, on average, 6.9 (SD = 2.5, *n* = 19), the systematic exposure component as 6.3 (SD = 2.9, *n* = 18), and health promotion as 7.3 (SD = 2.5, *n* = 19) (Table 2).

### Feasibility outcome 6: adverse events and negative experiences

Two participants (2/36, 6%) reported an adverse event each. One was enrolled in behavioral activation and systematic exposure and reported experiencing a sense of failure and despair due to being unable to engage with the treatment. The other was enrolled in systematic exposure and healthy lifestyle promotion and reported temporarily worsened mood as an effect of exposure. No participant reported more than one adverse event. No serious adverse event leading to permanent injury or an immediate need for clinical intervention was reported. On the NEQ-20, which measures negative effects of treatment, the proportion of endorsed items was 17% for increased symptoms, 0% for stigma, 3% for dependency on the treatment, 8% for hopelessness, and 11% for perceived insufficient quality of the treatment.

### Feasibility outcome 7: average within-group improvement

While preliminary efficacy was not the main focus of this feasibility study, we hoped to see at least moderate average reductions in key symptom domains across all participants in order to regard future work with these treatment components as worthwhile. Over the treatment period, there was a large mean reduction in depressive symptoms (PHQ-9, *d* = 0.83) and moderate reductions in general anxiety (GAD-7, *d* = 0.55) and fear of recurrence (FCRI-9, *d* = 0.54). Results were similar when analyzing only participants with clinically significant symptoms at baseline and excluding the attention/engagement group. Effects appeared to be at least maintained at the 3-month follow-up. See Online Resource 7 for details.

## Discussion

This study evaluated the feasibility of a randomized factorial study design for determining the unique contribution of specific treatment components to the total efficacy of an internet-delivered psychological treatment of long-term psychiatric symptoms in cancer survivors. Overall, we found the randomized factorial study design to be feasible in that therapists were able to manage participants receiving different combinations of components and approved of most key study procedures. An important finding, though unsurprising, was that interest from male cancer survivors was low. For the proposed trial design, there were also challenges related to the attention/engagement control group. For therapists, working with these participants led to some degree of ethical stress, and participants in this condition rated credibility as low. In other respects, the interventions themselves and the online format were feasible, with participants rating the treatments as credible and satisfactory, and showing adherence to the different treatment protocols. On average, participants reported a large reduction in depression symptoms and moderate reductions in general anxiety and fear of cancer recurrence, without serious adverse events.

Interest from male cancer survivors in participating in the trial was low. This is likely partly due to testicular and male thyroid cancer being uncommon compared to female breast cancer [62]. However, men are also less likely than women to use mental health services in general [63] and are underrepresented in trials of psychotherapy and lifestyle interventions for depression [64]. A more gender-targeted recruitment has been suggested to increase male participation in trials, which could be considered for a full-scale study [64].

A recurring theme during supervision was that therapists were uncertain about what information could be shared with participants in the attention/engagement control group, for example, in response to direct questions about managing certain types of mood disturbances. Though not an unusual practice in clinical trials [50], not offering interventions beyond attention/engagement sometimes led to ethical stress and discontent related to delivering a perceived less effective intervention. Some of these issues could likely be alleviated by providing a clearer framework for therapists regarding the attention/engagement treatment arm, or by removing or reducing the therapist-guided element of the intervention. Yet another alternative would be not to include an attention/engagement control group in a reduced factorial design [30]. It would still be possible to analyze the outcome of a randomized factorial trial in such a study, though the focus would be on the added effect of each specific treatment component over the *average* treatment, rather than on differences versus a control group [30].

Participants’ adherence to the treatment protocol was satisfactory, although the average number of days devoted and exercises conducted per week for the different treatment components would optimally be higher. Strategies for increasing engagement could be employed in a full-scale study. The proportion of participants reporting the online measurement strategy as very burdensome was also slightly higher than the preregistered target. A reduction in the number of questionnaires will therefore be considered for the full trial. Free text answers to the blinding check questions at the 3-month follow-up were also often difficult to interpret. These questions could perhaps be reformulated to result in clearer answers regarding possible unblinding.

## Clinical implications

Several randomized factorial trials have been conducted to assess internet-delivered psychological treatments for depression and anxiety [50]. However, these trials have typically evaluated aspects of the treatment format, for example, therapist-guided vs unguided, and have rarely evaluated treatment components themselves [50]. The randomized factorial study design evaluated here is therefore an innovative method, especially in psycho-oncology. In a full-scale trial, the contribution of each treatment component and their interactions will be modelled in the same statistical model, and the added effect of each treatment component to the overall treatment effect can be estimated. Psychiatric symptoms are common in cancer survivors [8, 9], and a full-scale factorial trial could provide the knowledge needed to develop an effective and evidence-based internet-delivered intervention, with increased access as a result of the online format. As the IN-FACT-0 trial has demonstrated the feasibility of the study design, it will form the basis of the design of a future full-scale factorial study, the IN-FACT-1 trial. It is also possible that this design could be used in evaluating complex therapies for other indications.

## Study limitations

Limitations of this study included the self-selected sample, which is highly likely to differ from samples drawn from routine clinical settings in several regards. Most participants were highly educated and worked full time, and only 8% were male. The feasibility of the interventions in groups with lower levels of education and functioning, and to some degree among males, is therefore less clear. On the other hand, women and highly educated individuals are also likely more inclined to show interest when psychological support is offered in routine care [65].

This study assessed several feasibility outcomes using a relatively small sample. This, although standard practice in feasibility studies, implies substantial uncertainty about individual findings as these were susceptible to variation due to mere sampling. Results should therefore be interpreted with caution. One measure of adherence used was also a retrospective post-treatment rating by participants of their adherence during the first and second half of the support program, where answers could have been affected by recall bias. To reduce recall bias, the assessment of adherence during the first half of treatment could be moved to the end of the fourth module. However, the study also included the number of initiated modules as a measure of adherence that is not affected by recall bias.

Preliminary efficacy outcomes were analyzed and indicated that there were moderate to large reductions in psychiatric symptoms. Within the feasibility framework, our line of reasoning was that should this study not be indicative of at least moderate within-group effects, it would not be feasible to study the treatment protocol further and proceed to test for causal effects in a full-scale randomized factorial trial. Given this aim and the small number of participants, results regarding preliminary efficacy must be interpreted with caution, and point estimates cannot reliably be compared with results from other studies. Substantially larger samples are needed to quantify how much specific components contribute to overall treatment effects, which would be the purpose of a full-scale randomized factorial trial.

## Conclusion

 A randomized factorial trial of the unique contribution of specific treatment components to an internet-delivered psychological treatment of long-term distress in cancer survivors appears to be feasible. This study design may be useful also for the development and evaluation of other complex interventions within psycho-oncology.

## Supplementary Information

Below is the link to the electronic supplementary material.

ESM 1 (PDF 2.76 MB)

## Data Availability

Individual participant data from this trial are not publicly available due to Swedish and European Union (EU) data protection and privacy legislation. Reasonable requests may be directed to the corresponding author, and will be considered on a case-by-case basis, with the assistance of juridical expertise and in accordance with the local guidelines of the sponsor. The trial protocol is available at ClinicalTrials.gov (NCT06046573).
