# Supplementary material for: Proof-of-concept for a randomized factorial design strategy to build a complex online support program for cancer survivors with psychiatric symptoms: the IN-FACT-0 feasibility trial
Source: J Cancer Surviv. 2025 Oct 25;20(5):2159–71. doi: 10.1007/s11764-025-01905-2 (PMC13723499; doi:10.1007/s11764-025-01905-2)
Supplement: Supplementary file 1 [file 11764_2025_1905_MOESM1_ESM.pdf]

Journal of Cancer Survivorship

Proof-of-concept for a randomized factorial design strategy to build a complex online support program for cancer survivors with psychiatric symptoms: the IN-FACT-0 feasibility trial

Julia Winter, Nils Gasslander, Gustaf Rane, Felicia Sundström, Linda Björkhem Bergman, Christel Hedman, Erland Axelsson

Supplementary Information

Online Resource 1. *Determination of sample size*……………………………………………………………………2

Online Resource 2. *Description of therapists*……………………………………………………………………3

Online Resource 3. *Overview of treatment protocols*……………………………………………………………4

Online Resource 4. *Overview of administered validated self-report questionnaires*……………………………8

Online Resource 5. *Psychiatric diagnoses at baseline based on structured telephone interviews*……………11

Online Resource 6. *Post-treatment assessment of blinding*……………………………………………………12

Online Resource 7. *Preliminary efficacy outcomes*……………………………………………………………14

Corresponding author: Julia Winter, Department of Molecular Medicine and Surgery, Karolinska Institutet, 171 76 Stockholm, Sweden, julia.winter@ki.se

Online Resource 1

Determination of sample size

The aim was to recruit 48 participants, a multiplier of eight, to allow for a cell size of six in each of the eight treatment arms. This would allow for two-sided mean tests of moderate ($d \approx 0.45$) within-group mean changes in efficacy outcomes with 80% power, given a 5% alpha and up to 20% missing data post-treatment. However, in light of such tests not being the primary focus of this feasibility study, unexpectedly little interest from testicular cancer survivors of whom we initially aimed to recruit 12, power in linear mixed models on repeated measurements being higher, and also time constraints, the decision was taken to terminate recruitment with 40 participants enrolled, resulting in a final cell size of five.

Online Resource 2

Description of therapists

Therapist	Profession
A	Licensed clinical psychologist
B	Licensed physician
C	Licensed clinical psychologist
D	Licensed clinical psychologist
E	MSc-level psychologist student

Online Resource 3

Overview of treatment protocols

Module	Attention/Engagement/Shared Content	Behavioral Activation	Systematic Exposure	Healthy Lifestyle Promotion
**1**	Content: Introduction to the treatment, the format and online platform. Instructions for setting meaningful goals for the treatment. Overview of the purpose of tracking wellbeing in a diary.<br><br>Key worksheets/fact sheets*:<br>• Set goals for your treatment<br>• Track your wellbeing (diary)	Content: Overview of depressive symptoms and fatigue as common negative effects of cancer and their causes. Introduction to the concept of behavioral activation and activity planning where behavioral activation aims to increase engagement in adaptive, goal-directed, and rewarding activities - i.e., positively reinforced behavior - while reducing avoidance and withdrawal behaviors - i.e., negatively reinforced behavior - that maintain or worsen unwanted emotional responses.<br><br>Key worksheets/fact sheets*:<br>• Activity diary**	Content: Overview of common negative effects of cancer such as fear of recurrence, cancer-related trauma, changes in the body and persistent symptoms. Registering and creating an overview of the participants own problems after cancer. Introduction to a standalone mindfulness exercise where participants unconditionally observed their own body.<br><br>Key worksheets/fact sheets*:<br>• Common problems after cancer<br>• To be with your body (mindfulness exercise)	Content: Overview of the importance of healthy living habits and how they can affect wellbeing after cancer. Introduction to making sustainable lifestyle changes.<br><br>Key worksheets/fact sheets*:<br>• Set goals for your treatment
**2**	Content: Text encouraging making long-term changes and regularly updating goals.<br><br>Key worksheets/fact sheets*:<br>• Track your wellbeing (diary)	Content: On positive and negative activities. Activity planning to increase positive activities and decrease negative activities as a way to reduce depressive symptoms and fatigue and improve wellbeing after cancer.<br><br>Key worksheets/fact sheets*:<br>• Evaluate positive and negative activities	Content: Expanding on the purpose of the mindfulness exercise. Introduction to safety behaviors and avoidance. The benefits of reducing safety behaviors in relation to negative effects after cancer.<br><br>Key worksheets/fact sheets*:<br>• To be with your body (mindfulness exercise)	Content: Benefits of physical activity and recommendations regarding physical activity.<br><br>Key worksheets/fact sheets*:<br>• Set goals for physical activity

		• Activity plan**	• Refraining from safety behaviors	
**3**	Content: Short text encouraging making changes.<br><br>Key worksheets/fact sheets*:<br>• Track your wellbeing (diary)	Content: Follow-up and analysis of the previous week's positive and negative activities. Suggestions for reducing negative activities. Advice for handling obstacles in activity planning. Overview of the ABC model (functional analysis) and how it can be applied to understand patterns of behavior through positive and negative reinforcement.<br><br>Key worksheets/fact sheets*:<br>• Activity plan**<br>• Use the ABC model	Content: Follow-up of the previous week's introduction to reducing safety behaviors. Introduction to systematic exposure and how it may be used to decrease negative effects after cancer. Practicing exposure as a means of reducing bodily discomfort.<br><br>Key worksheets/fact sheets*:<br>• To be with your body (mindfulness exercise)<br>• Refraining from safety behaviors<br>• Exposure for bodily signals	Content: Benefits of healthy eating habits and recommendations regarding nutrition.<br><br>Key worksheets/fact sheets*:<br>• Set goals for eating habits
**4**	Content: Short text encouraging reflecting on obstacles to making changes.<br><br>Key worksheets/fact sheets*:<br>• Track your wellbeing (diary)	Content: Follow-up of ABC model and how it may be used to optimize behavioral activation. Example of how the ABC-model is used by a fictional cancer survivor to understand negative patterns of behavior that contribute to maintaining negative symptoms such as fatigue.<br><br>Key worksheets/fact sheets*:<br>• Activity plan**	Content: Follow-up of the previous week's exercise to practice exposure in the presence of bodily discomfort. Advice to optimize the effects of exposure. Instructions to create own exposure exercises with examples from fictional cancer survivors. Follow-up of mindfulness exercise and encouragement to continue if the participant has found the exercise to be useful.<br><br>Key worksheets/fact sheets*:<br>• Plan and evaluate exposure<br>• Refraining from safety behaviors	Content: Importance of sleep and recommendations regarding sleep. Follow up of physical activity and healthy eating habits.<br><br>Key worksheets/fact sheets*:<br>• Set goals for sleep

**5**	Content: Short, encouraging text, including reflecting over how any changes made may be affecting wellbeing.<br><br>Key worksheets/fact sheets*:<br>• Track your wellbeing (diary)	Content: On handling obstacles in activity planning. Recommendations to regularly update and adjust the activity plan. The importance of meaningful activities and how the participant can identify what they find meaningful.<br><br>Key worksheets/fact sheets*:<br>• Activity plan**	Content: Follow-up of reducing safety behaviors and how to set appropriate goals. Follow-up of exposure exercises with encouragement to conduct exposure exercises daily, the importance of repetition and choosing appropriately challenging exercises. Using exposure to reduce anxiety and worry. Using mindfulness concepts to reduce catastrophic thoughts. Instructions to practice exposure for catastrophic thoughts by writing a story about the catastrophic thought. Examples of how fictional cancer survivors may continue working with exposure exercises.<br><br>Key worksheets/fact sheets*:<br>• Plan and evaluate exposure<br>• Refraining from safety behaviors	Content: Reducing alcohol consumption and smoking.<br><br>Key worksheets/fact sheets*:<br>• Set goals for reducing unhealthy habits
**6**	Content: Short text regarding the long-term perspective of making changes to improve wellbeing. Encouragement to reflect on goals set at the beginning of the treatment, what changes have been made to reach them, and what further changes are needed.<br><br>Key worksheets/fact sheets*:<br>• Track your wellbeing (diary)	Content: Instructions to continue with activity planning.<br><br>Key worksheets/fact sheets*:<br>• Activity plan**	Content: Instructions to continue reducing safety behaviors and continue with exposure exercises.<br><br>Key worksheets/fact sheets*:<br>• Plan and evaluate exposure<br>• Refraining from safety behaviors	Content: Instructions to continue to work towards healthy lifestyle changes.<br><br>Key worksheets/fact sheets*:<br>• Set goals for your treatment
**7**	Content: Short, encouraging text to continue with meaningful changes and normalizing obstacles.	Content: Instructions to continue with activity planning.	Content: Instructions to continue reducing safety behaviors and continue with exposure exercises.	Content: Instructions to continue to work towards healthy lifestyle changes.

	Key worksheets/fact sheets*:<br>• Track your wellbeing (diary)	Key worksheets/fact sheets*:<br>• Activity plan**	Key worksheets/fact sheets*:<br>• Plan and evaluate exposure<br>• Refraining from safety behaviors	Key worksheets/fact sheets*:<br>• Set goals for your treatment
**8**	Content: Information about continuing work with the treatment strategies to maintain progress and continue improvement. Advice for managing setbacks and instructions for making a plan for future setbacks.<br><br>Key worksheets/fact sheets*:<br>• Track your wellbeing (diary)<br>• Plan for setbacks	Content: Instructions to continue with activity planning.<br><br>Key worksheets/fact sheets*:<br>Activity plan**	Content: Instructions to continue reducing safety behaviors and continue with exposure exercises.<br><br>Key worksheets/fact sheets*:<br>• Plan and evaluate exposure<br>Refraining from safety behaviors	Content: Instructions to continue to work towards healthy lifestyle changes.<br><br>Key worksheets/fact sheets*:<br>Set goals for your treatment

*Participants were able to access all worksheets from previous modules.

**Participants receiving the behavioral activation component tracked their activities and wellbeing in the activity diary/plan. All other participants instead tracked wellbeing in a more simplified diary.

Online Resource 4

Overview of administered validated self-report questionnaires

Questionnaire	Outcome	Reference	SN	PRE	WK	PST	3MFU
Screening and safety only							
Alcohol Use Disorders Identification Test (AUDIT)	Alcohol use	(1)	x				
Drug Use Disorders Identification Test (DUDIT)	Drug use	(2)	x				
Hospital Anxiety and Depression Scale (HADS)	General distress	(3)	x				
Montgomery-Åsberg Depression Rating Scale item 9	Suicidal ideation	(4)	x	x	x	x	
Symptom domains and similar							
36-Item Short Form Health Survey (SF-36)	Health-related quality of life	(5)		x		x	
Generalized Anxiety Disorder 7 (GAD-7)	General anxiety	(6)	x	x	x	x	x
Patient Health Questionnaire-9 (PHQ-9)	Depression symptoms	(7)	x	x	x	x	x
9-item Fear of Cancer Recurrence Inventory (FCRI-9)	Fear of cancer recurrence	(8)	x	x	x	x	x
14-item Health Anxiety Inventory (HAI-14)	Health anxiety	(9)		x		x	x
Somatic Symptom Scale 8 (SSS-8)	Somatic symptom burden	(10)		x		x	x
Body Image Scale (BIS)	Body image	(11)	x			x	
12-item WHO Disability Assessment Schedule 2.0 (WD2-12)	Disability	(12)	x	x		x	x
Process and target variables							
3-item Behavioral Activation for Depression Scale – Activation (BADS-AC-3)	Behavioral activation	(13)		x	x	x	x
Symptom preoccupation scale (SYMPS)	Symptom preoccupation	Not yet published		x	x	x	x
Godin-Shephard Leisure-Time Physical Activity Questionnaire (GSLTPAQ)	Physical activity	(14)		x	x	x	x
Adapted self-efficacy scale	Self-efficacy	(15)		x	x	x	x
Credibility/Expectancy scale (C/E scale)	Credibility and expectancy	(16)			w.2		
Working Alliance Inventory (WAI) (6 items)	Relationship with the therapist	(17, 18)			w.2		
Explicit evaluation of therapy							
8-item Client satisfaction questionnaire (CSQ-8)	Satisfaction with treatment	(19)				x	
20-item Negative Effects Questionnaire (NEQ-20)	Negative effects of treatment	(20)				x	
Cost-effectiveness outcomes							
EuroQol 5D (EQ5D)	Utility score	(21)		x		x	x
Trimbos questionnaire (TIC-P)	Resource use	(22)		x		x	x

SN=screening, PRE=pre-treatment, WK=weekly, PST=post-treatment, 3MFU=three-month follow-up, w.2=administered at the second weekly assessment during treatment.

References

1. Saunders JB, Aasland OG, Babor TF, de la Fuente JR, Grant M. Development of the Alcohol Use Disorders Identification Test (AUDIT): WHO Collaborative Project on Early Detection of Persons with Harmful Alcohol Consumption - II. Addiction. 1993;88(6):791-804.
2. Berman AH, Bergman H, Palmstierna T, Schlyter F. Evaluation of the Drug Use Disorders Identification Test (DUDIT) in criminal justice and detoxification settings and in a Swedish population sample. Eur Addict Res. 2005;11(1):22-31.
3. Norton S, Cosco T, Doyle F, Done J, Sacker A. The Hospital Anxiety and Depression Scale: a meta confirmatory factor analysis. J Psychosom Res. 2013;74(1):74-81.
4. Svanborg P, Åsberg M. A new self-rating scale for depression and anxiety states based on the Comprehensive Psychopathological Rating Scale. Acta Psychiatr Scand. 1994;89(1):21-8.
5. Persson LO, Karlsson J, Bengtsson C, Steen B, Sullivan M. The Swedish SF-36 Health Survey II. Evaluation of clinical validity: results from population studies of elderly and women in Gothenborg. J Clin Epidemiol. 1998;51(11):1095-103.
6. Kroenke K, Spitzer RL, Williams JB, Monahan PO, Lowe B. Anxiety disorders in primary care: prevalence, impairment, comorbidity, and detection. Ann Intern Med. 2007;146(5):317-25.
7. Kroenke K, Spitzer RL, Williams JB. The PHQ-9: validity of a brief depression severity measure. J Gen Intern Med. 2001;16(9):606-13.
8. Simard S, Savard J. Screening and comorbidity of clinical levels of fear of cancer recurrence. J Cancer Surviv. 2015;9(3):481-91.
9. Salkovskis PM, Rimes K, Warwick H, Clark D. The Health Anxiety Inventory: development and validation of scales for the measurement of health anxiety and hypochondriasis. Psychol Med. 2002;32(5):843-53.
10. Hybelius J, Kosic A, Salomonsson S, Wachtler C, Wallert J, Nordin S, et al. Measurement Properties of the Patient Health Questionnaire-15 and Somatic Symptom Scale-8: A Systematic Review and Meta-Analysis. JAMA Netw Open. 2024;7(11):e2446603.
11. Hopwood P, Fletcher I, Lee A, Al Ghazal S. A body image scale for use with cancer patients. Eur J Cancer. 2001;37(2):189-97.
12. Axelsson E, Lindsäter E, Ljótsson B, Andersson E, Hedman-Lagerlöf E. The 12-item Self-Report World Health Organization Disability Assessment Schedule (WHODAS) 2.0 Administered Via the Internet to Individuals With Anxiety and Stress Disorders: A Psychometric Investigation Based on Data From Two Clinical Trials. JMIR Ment Health. 2017;4(4):e58.
13. Axelsson E, Santoft F, Särnholm J, Ljótsson B. Brief scales for the measurement of target variables and processes of change in cognitive behavior therapy for major depression, panic disorder, and social anxiety disorder. Submitted.
14. Amireault S, Godin G, Lacombe J, Sabiston CM. The use of the Godin-Shephard Leisure-Time Physical Activity Questionnaire in oncology research: a systematic review. BMC Med Res Methodol. 2015;15:60.
15. Lomi C, Nordholm LA. Validation of a Swedish version of the Arthritis Self-efficacy Scale. Scand J Rheumatol. 1992;21(5):231-7.
16. Devilly GJ, Borkovec TD. Psychometric properties of the credibility/expectancy questionnaire. J Behav Ther Exp Psychiatry. 2000;31(2):73-86.
17. Hatcher RL, Gillaspy JA. Development and validation of a revised short version of the Working Alliance Inventory. Psychother Res. 2006;16(1):12-25.
18. Hedman E, Axelsson E, Gorling A, Ritzman C, Ronnheden M, El Alaoui S, et al. Internet-delivered exposure-based cognitive-behavioural therapy and behavioural stress management for severe health anxiety: randomised controlled trial. Br J Psychiatry. 2014;205(4):307-14.
19. Kelly PJ, Kyngdon F, Ingram I, Deane FP, Baker AL, Osborne BA. The Client Satisfaction Questionnaire-8: Psychometric properties in a cross-sectional survey of people attending residential substance abuse treatment. Drug Alcohol Rev. 2018;37(1):79-86.

20. Rozental A, Kottorp A, Forsstrom D, Mansson K, Boettcher J, Andersson G, et al. The Negative Effects Questionnaire: psychometric properties of an instrument for assessing negative effects in psychological treatments. Behav Cogn Psychother. 2019;47(5):559-72.
21. EuroQol Group. EuroQol - a new facility for the measurement of health-related quality of life. Health Policy. 1990;16(3):199-208.
22. Hakkaart-van Roijen L, Van Straten A, Donker M, Tiemens B. Trimbos/iMTA questionnaire for costs associated with psychiatric illness (TIC-P). Rotterdam: Institute for Medical Technology Assessment, Erasmus University Rotterdam; 2002.

Online Resource 5

Psychiatric diagnoses at baseline based on structured telephone interviews

Psychiatric diagnoses	*n* (%)
Major depressive disorder	16 (40.0)
Panic disorder with or without agoraphobia	0 (0.0)
Agoraphobia (without panic disorder)	1 (2.5)
Social anxiety disorder	1 (2.5)
Obsessive-compulsive disorder	1 (2.5)
Posttraumatic stress disorder	2 (5.0)
Alcohol use disorder	0 (0.0)
Substance use disorder	0 (0.0)
Eating disorder	1 (2.5)
Generalised anxiety disorder	7 (17.5)
Clinically significant health anxiety	20 (50.0)
Other	1 (2.5)

Online Resource 6

Post-treatment assessment of blinding

This post-treatment assessment of blinding was administered as part of the three-month follow-up. Participants in the attention/engagement treatment arm (control group) did not answer the three-month follow-up due to being crossed over. Answers were available from 31/35 (89%) participants. Participants who answered “No” to question 1 did not complete the following questions.

In this study you have been randomly assigned to one of several different support programs.
(“I denna studie har du lottats till ett av flera olika stödprogram.”)

1. Do you know which these support programs were?
   (“1. Vet du vilka dessa stödprogram var?)

	n (%)
Yes (”Ja”)	6 (19)
No (”Nej”)	14 (45)
Unsure (”Osäker”)	11 (36)

2. Describe with a few words what the support programs are called or what they entail.
   (“2. Beskriv med några få ord vad stödprogrammen kallas eller vad de går ut på.”)
   If you answered “Unsure” to the above question, explain why and guess what the support programs were.
   (“Om du svarade ”Osäker” på frågan ovan, förklara varför och gissa vilka stödprogrammen var.”

	n
Blinded (description not accurate)	13
Possibly unblinded (rudimentary description of treatments, unclear answer or no answer)	4

3. Do you know with certainty which support programs the researchers believed would be most effective, for example, because this was revealed during the study?
(“Vet du med säkerhet vilka typer av stödprogram forskarna trodde skulle vara mest effektiva, till exempel för att det avslöjades under studien?)

	Blinded according to item 2	Possibly unblinded according to item 2
	n	*n*
Yes (”Ja”)*	2*	1*
No (”Nej”)	11	3

*The “yes” answer option was accompanied by a text box with the following prompt: The researchers believed the following types of support programs would be most effective: (“Forskarna trodde att följande typer av stödprogram skulle vara mest effektiva:”). No participants who answered “yes” made reference to any treatment component by name or to the factorial nature of the study.

Online Resource 7

Preliminary efficacy outcomes

		Pre-treatment			Post-treatment			3MFU[e]			Change Pre-Post[f]			Change Post-3MFU[eg]		
Outcome (measure, theoretical range)	Treatment group	*n*	M	SD	*n*	M	SD	*n*	M	SD	Slope	(95% CI)	Cohen's *d*[h]	Slope	(95% CI)	Cohen's *d*[h]
Depressive symptoms (PHQ-9, 0-27)[a]	Total	40	8.8	4.7	36	4.3	3.7	31	3.9	3.2	-3.9	(-4.8--3.1)	0.83			
	Clin sign[c]	20	11.1	4.5	17	4.4	4.1	17	4.5	3.9	-5.7	(-6.9--4.4)	1.28	-0.1	(-1.7-1.4)	0.03
	BA	20	8.6	4.1	19	3.8	2.3	18	3.9	2.7	-4.5	(-5.5--3.5)	1.09	0.1	(-1.2-1.3)	0.02
	EXP	20	8.7	4.4	18	4.3	4.2	17	4.6	3.9	-4.3	(-5.4--3.3)	0.97	0.6	(-0.7-1.8)	0.13
	HLP	20	8.0	4.8	19	3.1	2.6	18	3.4	2.5	-4.2	(-5.1--3.2)	0.86	0.8	(-0.4-1.9)	0.16
	A/E only	5	9.2	5.7	4	8.3	4.2				-1.5	(-4.8-1.8)	0.26			
	1 component	15	9.3	5.3	13	4.4	4.6	13	3.8	3.7	-3.5	(-5.0--2.0)	0.67	-0.8	(-2.6-1.1)	0.15
	2 components	15	8.8	4.4	14	2.9	2.1	14	4.1	3.0	-5.2	(-6.2--4.2)	1.18	1.3	(0.2-2.5)	0.30
	3 components	5	6.8	3.6	5	4.6	3.2	4	3.8	3.0	-3.3	(-5.2--1.4)	0.93	-0.1	(-2.5-2.3)	0.02
General anxiety (GAD-7, 0-21)[a]	Total	40	7.3	4.7	36	3.9	4.6	31	2.7	2.5	-2.6	(-3.5--1.7)	0.55			
	Clin sign[c]	16	10.4	4.7	13	6.2	6.3	12	3.9	2.6	-2.6	(-4.5--0.6)	0.55	-2.3	(-4.7-0.2)	0.48
	BA	20	6.6	4.8	19	3.9	4.1	18	2.6	2.3	-2.9	(-4.0--1.8)	0.60	-1.0	(-2.3-0.4)	0.21
	EXP	20	7.6	5.2	18	4.3	6.1	17	2.6	2.8	-2.6	(-3.8--1.4)	0.50	-1.0	(-2.5-0.5)	0.20
	HLP	20	6.7	4.5	19	2.9	3.6	18	2.8	2.8	-3.4	(-4.5--2.3)	0.76	0.4	(-1.0-1.7)	0.08
	A/E only	5	7.2	2.8	4	4.8	2.2				-0.8	(-3.2-1.6)	0.29			
	1 component	15	7.7	5.3	13	4.4	5.8	13	2.5	2.4	-1.7	(-3.5-0.0)	0.33	-2.1	(-4.2-0.0)	0.39
	2 components	15	8.2	5.0	14	3.4	3.9	14	3.1	2.7	-4.2	(-5.4--2.9)	0.84	0.1	(-1.4-1.6)	0.02
	3 components	5	3.6	1.3	5	3.8	5.5	4	1.8	2.9	-1.8	(-3.4--0.1)	1.32	-0.2	(-2.3-1.8)	0.18
Fear of recurrence (FCRI-9, 0-36)[a]	Total	40	21.9	5.1	36	18.6	5.5	31	17.3	5.6	-2.7	(-3.5--2.0)	0.54			
	Clin sign[c]	34	22.1	5.0	31	18.6	5.6	30	17.4	5.6	-3.0	(-3.8--2.2)	0.59	-0.7	(-1.6-0.3)	0.13
	BA	20	22.3	5.0	19	18.9	5.8	18	17.7	5.8	-3.0	(-4.0--2.1)	0.61	-0.8	(-2.0-0.4)	0.16
	EXP	20	22.8	5.3	18	18.9	6.8	17	17.3	7.0	-4.2	(-5.3--3.0)	0.79	-0.3	(-1.7-1.1)	0.06
	HLP	20	21.0	5.1	19	17.1	4.9	18	15.6	4.9	-2.9	(-4.0--1.9)	0.58	-1.0	(-2.3-0.2)	0.20
	A/E only	5	21.6	4.7	4	19.8	5.4				-0.7	(-2.5-1.2)	0.14			

	**1 component**	15	20.9	4.7	13	18.2	5.6	13	17.5	4.9	-1.8	(-3.0--0.7)	0.39	-0.6	(-2.0-0.8)	0.14
	**2 components**	15	23.4	6.0	14	19.6	4.7	14	18.7	5.5	-3.5	(-4.7--2.4)	0.59	0.1	(-1.3-1.5)	0.02
	**3 components**	5	20.4	2.7	5	16.0	8.2	4	11.8	6.0	-4.6	(-6.9--2.3)	1.70	-2.7	(-5.6-0.1)	1.01
**Physical HRQOL (PCS)[b]**	**Total**	40	44.4	8.9	36	47.9	9.7				3.3	(0.5-6.1)	0.37			
	**Total - A/E[d]**	35	43.2	8.8	32	47.2	10.0				3.6	(0.6-6.7)	0.41			
	**BA**	20	46.1	8.3	19	46.3	10.4				-0.1	(-2.7-2.6)	0.01			
	**EXP**	20	43.0	8.9	18	48.6	8.9				5.2	(0.6-9.9)	0.59			
	**HLP**	20	43.9	9.7	19	47.0	11.1				2.9	(-1.1-6.9)	0.30			
	**A/E only**	5	52.4	4.6	4	53.88	5.11				1.4	(-4.1-6.8)	0.30			
	**1 component**	15	39.0	6.4	13	45.7	9.2				6.5	(1.1-11.9)	1.02			
	**2 components**	15	47.0	9.5	14	49.7	10.7				2.4	(-2.2-7.0)	0.25			
	**3 components**	5	44.4	8.9	5	44.0	10.0				-0.3	(-6.9-6.2)	0.04			
**Mental HRQOL (MCS)[b]**	**Total**	40	32.8	11.6	36	41.1	11.7				7.8	(4.3-11.4)	0.68			
	**Total - A/E[d]**	35	33.8	11.5	32	41.7	11.4				7.7	(3.9-11.5)	0.67			
	**BA**	20	33.8	11.9	19	41.2	11.3				7.3	(2.1-12.5)	0.61			
	**EXP**	20	34.3	11.3	18	42.0	12.1				7.9	(2.3-13.4)	0.70			
	**HLP**	20	34.2	10.6	19	44.3	10.7				9.6	(5.0-14.3)	0.91			
	**A/E only**	5	25.6	10.8	4	36.5	14.6				8.5	(-6.0-23.0)	0.79			
	**1 component**	15	34.5	11.6	13	39.3	11.0				4.4	(-1.9-10.6)	0.38			
	**2 components**	15	31.5	12.7	14	43.1	12.5				11.5	(6.9-16.2)	0.91			
	**3 components**	5	38.7	5.3	5	44.2	10.1				5.4	(-4.7-15.6)	1.02			

[a]Measured prior to treatment, weekly during treatment, after treatment and three months after treatment.
[b]Health-related quality of life measured using two summary health component scores, the physical component summary (PCS) and mental component summary (MCS). Generated by aggregating and transforming results from the 36-Item Short Form Health Survey using Swedish normative data and previously established physical and mental factor score coefficients to a T score with a mean of 50 and a standard deviation of 10 (1, 2). A higher score indicates a higher health-related quality of life. Measured before and after treatment.
[c]Participants with clinically significant depressive symptoms, general anxiety and fear of recurrence at baseline, respectively, and not including participants in the attention/engagement arm (control group). Cut-offs for clinical significance were PHQ-9≥10, GAD-7≥8 and FCRI-9≥16.
[d]All participants excluding those in the attention/engagement arm.

[e]Participants in the attention/engagement arm (n=5) did not answer the three-month follow-up due to crossing over and results are therefore not shown for the total group at three months.
[f]Change in outcome from prior to treatment to after treatment.
[g]Change in outcome from after treatment to three months after treatment.
[h]Cohen's *d*, calculated with the model-implied mean change as the numerator and pre-treatment standard deviation as the denominator. A value of $d = 0.2$ was regarded as a small effect, $d = 0.5$ as a moderate effect, and $d = 0.8$ as a large effect.

PHQ-9, Patient Health Questionnaire 9; GAD-7, Generalized Anxiety Disorder 7; FCRI-9, 9-item Fear of Cancer Recurrence Inventory; HRQOL, health-related quality of life; PCS, Physical Component Summary; MCS, Mental Component Summary, A/E, attention/engagement; BA, behavioral activation; EXP, systematic exposure; HLP, healthy living promotion; 3MFU, three-month follow-up; M, mean; SD, standard deviation; CI, confidence interval

References

1. Maruish ME. User's manual for the SF-36v2 Health Survey (3rd ed.). Lincoln, RI: QualityMetric Incorporated; 2011.
2. Sullivan M, Karlsson J, Taft C. SF-36 Hälsoenkät: Svensk Manual och Tolkningsguide 2ed. Gothenburg: Sahlgrenska University Hospital; 2002.
